# Distinct Inflammatory Programming of Thoracic Cavity White Adipose Immune Cells Regulates Influenza Pathogenesis

**DOI:** 10.1093/infdis/jiag201

**Published:** 2026-04-07

**Authors:** Cassidy J Ulanowicz, Pablo C Alarcon, Michelle S M A Damen, Jennifer L Wayland, Keisuke Sawada, John Eom, Traci E Stankiewicz, Hak Chung, Kristin Lampe, Sara Szabo, Maria E Moreno-Fernandez, Nathan Salomonis, Senad Divanovic

**Affiliations:** Divisions of Immunobiology, Cincinnati Children's Hospital Medical Center, Cincinnati, Ohio, USA; Immunology Graduate Program, Cincinnati Children's Hospital Medical Center, Cincinnati, Ohio, USA; Divisions of Immunobiology, Cincinnati Children's Hospital Medical Center, Cincinnati, Ohio, USA; Immunology Graduate Program, Cincinnati Children's Hospital Medical Center, Cincinnati, Ohio, USA; Medical Scientist Training Program, University of Cincinnati College of Medicine, Cincinnati, Ohio, USA; Divisions of Immunobiology, Cincinnati Children's Hospital Medical Center, Cincinnati, Ohio, USA; Gastroenterology, Hepatology, and Nutrition, Cincinnati Children's Hospital Medical Center, Cincinnati, Ohio, USA; Divisions of Immunobiology, Cincinnati Children's Hospital Medical Center, Cincinnati, Ohio, USA; Immunology Graduate Program, Cincinnati Children's Hospital Medical Center, Cincinnati, Ohio, USA; Medical Scientist Training Program, University of Cincinnati College of Medicine, Cincinnati, Ohio, USA; Divisions of Immunobiology, Cincinnati Children's Hospital Medical Center, Cincinnati, Ohio, USA; Immunology Graduate Program, Cincinnati Children's Hospital Medical Center, Cincinnati, Ohio, USA; Medical Scientist Training Program, University of Cincinnati College of Medicine, Cincinnati, Ohio, USA; Divisions of Immunobiology, Cincinnati Children's Hospital Medical Center, Cincinnati, Ohio, USA; Divisions of Immunobiology, Cincinnati Children's Hospital Medical Center, Cincinnati, Ohio, USA; Divisions of Immunobiology, Cincinnati Children's Hospital Medical Center, Cincinnati, Ohio, USA; Gastroenterology, Hepatology, and Nutrition, Cincinnati Children's Hospital Medical Center, Cincinnati, Ohio, USA; Department of Pediatrics, University of Cincinnati College of Medicine, Cincinnati, Ohio, USA; Pathology, Cincinnati Children's Hospital Medical Center, Cincinnati, Ohio, USA; Immunology Graduate Program, Cincinnati Children's Hospital Medical Center, Cincinnati, Ohio, USA; Gastroenterology, Hepatology, and Nutrition, Cincinnati Children's Hospital Medical Center, Cincinnati, Ohio, USA; Department of Pediatrics, University of Cincinnati College of Medicine, Cincinnati, Ohio, USA; Immunology Graduate Program, Cincinnati Children's Hospital Medical Center, Cincinnati, Ohio, USA; Department of Pediatrics, University of Cincinnati College of Medicine, Cincinnati, Ohio, USA; Biomedical Informatics, Cincinnati Children's Hospital Medical Center, Cincinnati, Ohio, USA; Divisions of Immunobiology, Cincinnati Children's Hospital Medical Center, Cincinnati, Ohio, USA; Immunology Graduate Program, Cincinnati Children's Hospital Medical Center, Cincinnati, Ohio, USA; Medical Scientist Training Program, University of Cincinnati College of Medicine, Cincinnati, Ohio, USA; Department of Pediatrics, University of Cincinnati College of Medicine, Cincinnati, Ohio, USA; Center for Inflammation and Tolerance, Cincinnati Children's Hospital Medical Center, Cincinnati, Ohio, USA

**Keywords:** white adipose tissue, obesity, high-fat diet (HFD), inflammation, lymphocytes, T cells, B cells

## Abstract

**Background:**

Obesity-associated inflammation in white adipose tissue (WAT) worsens outcomes of influenza A virus (IAV) infection. A recently identified thoracic cavity WAT (tcWAT) supports IAV replication. However, tcWAT immune cell composition, functional properties, and role in IAV disease severity remain unclear.

**Methods:**

Using a mouse model of diet-induced obesity, flow cytometry, and single-cell RNA sequencing, we compared tcWAT with lung-distal visceral WAT assessing immune cell composition, transcriptomic profiles, inflammatory potential, and impact on IAV pathogenesis.

**Results:**

At baseline, tcWAT was uniquely enriched for immune cells with heightened proinflammatory capacity and exhibited a striking predominance of lymphocytic populations, including immature and Satb1^+^ T cells, the latter expressing gene signatures associated with elevated T-cell activation. Transfer of tcWAT immune cells into IAV-infected recipients accelerated IAV disease severity. IAV infection robustly reshaped tcWAT immune landscape, driving expansion of a B-cell population expressing Zbtb32 and upregulating genes involved in immune regulation and antiviral responses. Concurrently, IAV infection reduced immune populations linked to neutrophil regulation in tcWAT, while these same populations were expanded in the lung.

**Conclusions:**

These findings identify lung-proximal tcWAT as a distinct inflammatory tissue that may amplify pathogenic immune responses during IAV infection.

## Teaser

Thoracic cavity white adipose tissue (tcWAT) is immunologically unique compared to other visceral WAT depots. tcWAT immune cells may contribute to the exacerbated influenza disease severity in obesity.

Despite widespread vaccination and substantial advances in understanding influenza A virus (IAV) pathogenicity, IAV infections remain a major global public health concern with 3–5 million cases of severe illness reported annually [1]. IAV infection triggers an inflammatory response through activation of immune pathways and release of proinflammatory cytokines [2–4].

Obesity, characterized by excessive adiposity and chronic low-grade inflammation, affects nearly 1 billion individuals worldwide [5] and is an independent risk factor for severe pulmonary disease severity [6]. Individuals with obesity are more susceptible to IAV infection and experience worse disease outcomes [7–9]. Obesity skews immune cell function and is a contributor to amplified tissue pathology in IAV infection [7, 9–11].

Visceral white adipose tissue (WAT) serves as an energy reservoir, insulates organs [12], and contributes to endocrine communication [13]. Visceral WAT is composed of adipocytes, preadipocytes, fibroblasts, endothelial cells, and immune cells [14]. Expansion of visceral WAT (eg, perirenal [pWAT] and epidydimal [eWAT]), drives inflammation and immune dysfunction in obesity [15]. In mice, eWAT best models human visceral WAT [16] and represents the most extensively studied depot in obesity and influenza research [17–20].

IAV is detected in visceral WAT of lean mice [17, 18, 21, 22] where it upregulates antiviral pathways [18, 21]. IAV can also replicate in lean human adipocytes [23]. We previously demonstrated that IAV is detected in eWAT and that adipose tissue macrophages develop an antiviral phenotype [17]. Additionally, we showed that transcriptional signatures of lipid-associated macrophages are decreased in eWAT but increased in lungs of IAV-infected obese mice [17]. More recently, a distinct visceral depot surrounding the lungs and heart—thoracic cavity WAT (tcWAT)—was described [23]. Although IAV was detected in tcWAT [23], its immune landscape and functional role during IAV infection remained undefined. Here, we characterize tcWAT immune composition at baseline and during IAV infection, revealing tcWAT as a highly immunologically enriched and distinct visceral WAT depot that contributes to IAV-induced inflammation and disease severity in obesity.

## METHODS

Comprehensive methodology is in Supplementary Methods.

### Obesogenic Diet Model

C57BL/6J wild-type mice were housed and bred at Cincinnati Children's Hospital Medical Center in a specific-pathogen–free facility. Male mice, 6 to 8 weeks old, were fed an irradiated high-fat diet (HFD).

### Influenza Infections

Influenza infections were carried out as described [17, 24]. Mice were sedated via isoflurane inhalation and treated intranasally with saline (50 μL) or H1N1 PR8 influenza (30 hemagglutinin units in 50 μL).

### Quantitative Reverse Transcriptase-Polymerase Chain Reaction

WAT RNA was extracted and reverse-transcribed to cDNA. Gene expression was measured via quantitative polymerase chain reaction (qPCR). Primer sequences are listed in Supplementary Table 1.

### Immune Cell Isolation

Lung and WAT immune cells were isolated using enzymatic digestion as described [17, 24–26].

### Flow Cytometry

Lung and WAT immune cells were labeled with monoclonal antibodies (listed in Supplementary Table 2), fixed, and stimulated with Phorbol 12-myristate 13-acetate (PMA), ionomycin, and brefeldin A as described [17, 24, 25]. Data were collected with an LSR Fortessa and analyzed via FlowJo X.

### Histopathological Analysis

WAT sections were stained with hematoxylin and eosin. Histological scoring was performed by an experienced senior certified pathologist (S.S.). (Supplementary Figure 1).

### Single-Cell RNA Sequencing and Analysis

Single-cell RNA sequencing (scRNAseq) was performed on 5000–10 000 targeted CD45^+^ immune cells isolated by fluorescence-activated cell sorting (FACS) from tcWAT, eWAT, and lung using 10x Genomics protocols. Demultiplexed FASTQ files were processed with CellRanger to produce matrix files. MarkerGenes, Sankey plots, and differential expression between each scRNAseq condition was performed using AltAnalyze3. Marker gene set enrichment was performed with ToppFun. The scRNAseq data sets have been deposited in the Gene Expression Omnibus Database (accession GSE266326).

### Adoptive Transfers

Immune cells were isolated from WAT, enriched via FACS and transferred (5 × 10^5^ cells/mouse) into IAV-infected recipient mice as described [17].

### Statistical Analysis

Statistical analysis was completed using Prism 10 (GraphPad), including unpaired Student *t* test, a 1-way analysis of variance, and a 2-way analysis of variance. All values are represented as mean ± standard error of the mean (SEM). Only histological analyses/quantification were conducted in blinded fashion.

## RESULTS

### Immune Cell-Enriched Thoracic White Adipose Tissue Amplifies IAV-Induced Lung Inflammation

**Figure 1.**
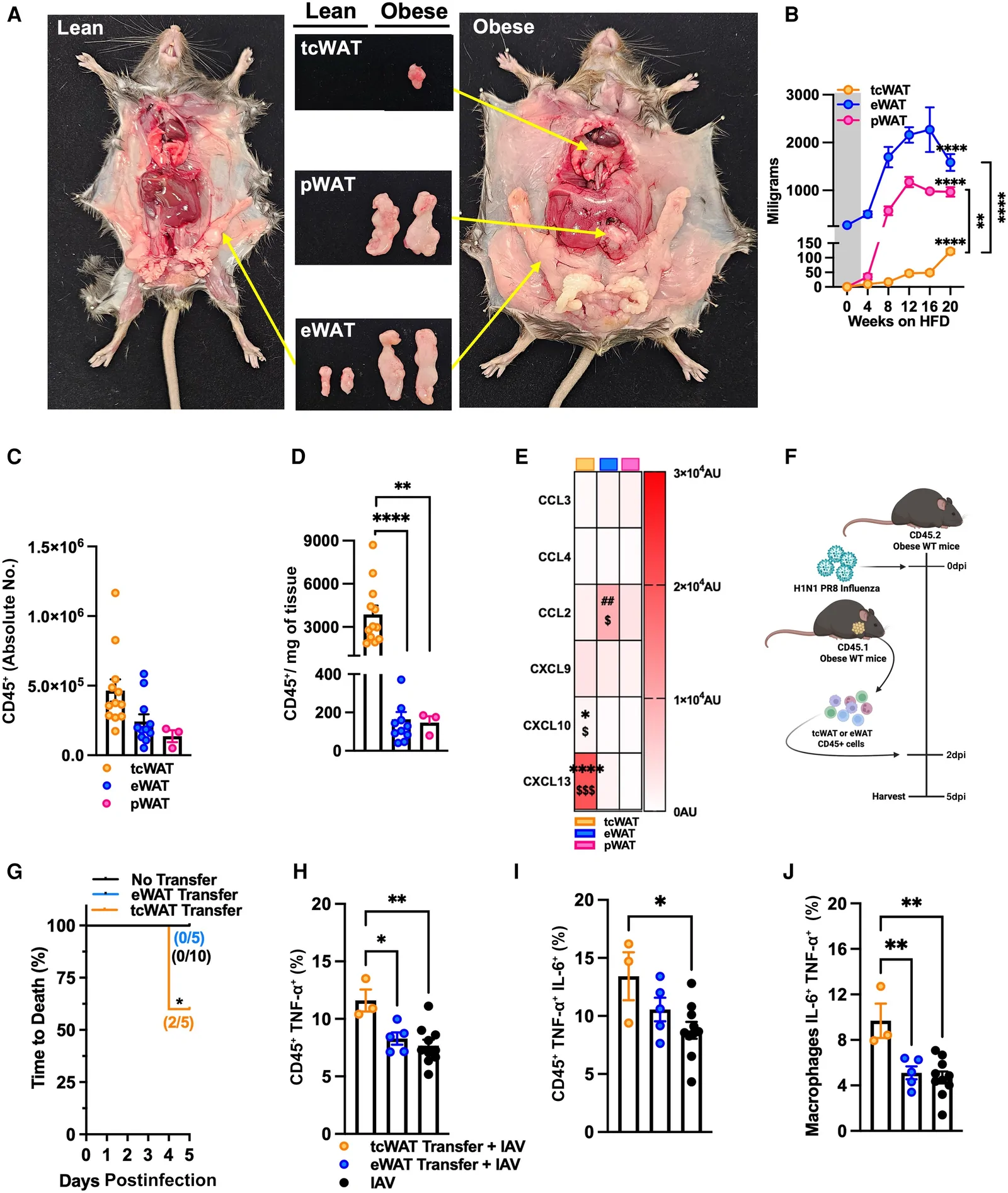
Thoracic cavity WAT exhibits heightened immune cell repertoire. Eight-week-old WT C57BL/6 mice were fed chow diet (fat 10%, carbohydrate 70%, protein 20%) or HFD (fat 60%, carbohydrate 20%, protein 20%; research diet D12492) for 4, 8, 12, 16, 20 weeks. *A*, Image of lean and obese mouse abdominal and thoracic cavity WAT depots. Yellow arrows from top to bottom pointing to tcWAT, pWAT and eWAT respectively. *B*, Weight of WAT depots over the course of HFD feeding. *C*, Absolute number of total CD45^+^ cells at 20 weeks of HFD across WAT depots. *D*, Absolute number of total CD45^+^ cells/mg of tissue at 20 weeks of HFD across WAT depots. *E*, qPCR analysis of various chemokine genes (*Cxcl3, Cxcl10, Cxcl9, Ccl2, Ccl3, Ccl4*) at 20 weeks of HFD across WAT depots. Values are reported as AU normalized to β-actin. *F*, Adoptive transfer design schematic: 8-week-old WT CD45.2-expressing C57BL/6 mice and CD45.1-expressing BoyJ mice were fed HFD for 30 weeks. CD45.2-expressing C57BL/6 mice were subsequently intranasally infected with IAV (A/PR/8/34 [H1N1], batch 4XP160913, 30 hemagglutinin units; Charles River). At 2 dpi, tcWAT or eWAT immune cells were isolated from BoyJ mice and adoptively transferred via tail-vein injection into IAV-infected CD45.2-expressing recipient mice (n = 3–10/group, combined result of 2 independent experiments). *G*, Time to death throughout the course of infection. *H*–*J*, Flow cytometry data of host-originated lung macrophages posttransfer at 5 dpi: (*H*) total CD45^+^ cells TNF^+^; (*I*) total CD45^+^ cells TNF^+^ and IL-6^+^; (*J*) macrophages IL-6^+^ and TNF^+^. All data are presented as mean ± SEM. *B*, Two-way analysis of variance (Turkey). *C–E, H–J*, One-way analysis of variance (Dunnett). *G*, Simple survival analysis (Kaplan-Meier). Sample size: (*B*–*D*) n = 3–12/group, combined result of 2 independent experiments; (*E*) n = 5–9/group, combined result of 6 independent experiments; (*G–J*) n = 3–10, combined result of 2 independent experiments. Statistical significance levels: (*B–D*, *G–J*) *P* < .05, **P < .01, ****P < .0001; (*E*) * significant difference from eWAT; $: significant difference from pWAT; # significant difference from tcWAT. Abbreviations: AU, arbitrary unit; dpi, days postinfection; eWAT, epidydimal WAT; IAV, influenza A virus; IL-6, interleukin 6; pWAT, perirenal WAT; qPCR, quantitative polymerase chain reaction; tcWAT, thoracic cavity WAT; TNF, tumor necrosis factor; WAT, white adipose tissue; WT, wild type.

To investigate obesity-dependent inflammatory changes throughout visceral WAT development, eWAT, pWAT, and tcWAT depots of lean and obese mice were characterized (Figure 1*A*). As expected, obesity caused temporal expansion of eWAT and pWAT (Figure 1*B*). Notably, obesity also produced a small, unilobular tcWAT depot (Figure 1*A* and 1*B*). Surprisingly, despite being significantly smaller, tcWAT exhibited robust immune cell accrual (Supplementary Figure 2*A*–*C*, left) and density throughout obesity development (Supplementary Figure 2*A*–*C*, right). Despite differences in size, total immune cell numbers were similar across all WAT depots after 20 weeks on HFD (Figure 1*C*). However, tcWAT immune cell density was approximately 10-fold higher per tissue weight compared to eWAT and pWAT (Figure 1*D*). Given heightened immune cell abundance in tcWAT, chemokine gene expression in visceral WAT was analyzed via qPCR. Compared to other depots, tcWAT exhibited amplified *Cxcl10* and *Cxcl13* expression, strong chemoattractants for T cells and B cells, respectively [27, 28], and decreased *Ccl2* expression, strong chemoattractant for monocytes and macrophages [29] (Figure 1*E*).

Proximity to the lung and immune cell enrichment led us to investigate if tcWAT immune cells play a role in IAV disease severity, using an IAV dose that drives mortality in obese mice by 6 days postinfection (6 dpi) [24]. Adoptive transfer of tcWAT immune cells (CD45^+^) from uninfected obese donor mice into IAV-infected recipient mice (Figure 1*F*) led to accelerated time to death compared to transfer of eWAT immune cells or mock-transfer controls (Figure 1*G*). Of note, other influenza disease severity parameters (weight loss and lung histopathology) were similar between groups (Data not shown). The observed increase in mortality positively correlated with significant increases in the hosts’ lung total immune cell tumor necrosis factor (TNF) production (Figure 1*H*), total immune cell TNF and interleukin 6 (IL-6) coproduction (Figure 1*I*), and hosts’ lung macrophage TNF and IL-6 coproduction (Figure 1*J*). These data suggest obesity promotes emergence of an immunogenic tcWAT depot, which may foster an inflammatory environment that contributes to pathogenic outcomes during IAV infection.

### Thoracic White Adipose Tissue Has a Uniquely Proinflammatory Profile

**Figure 2.**
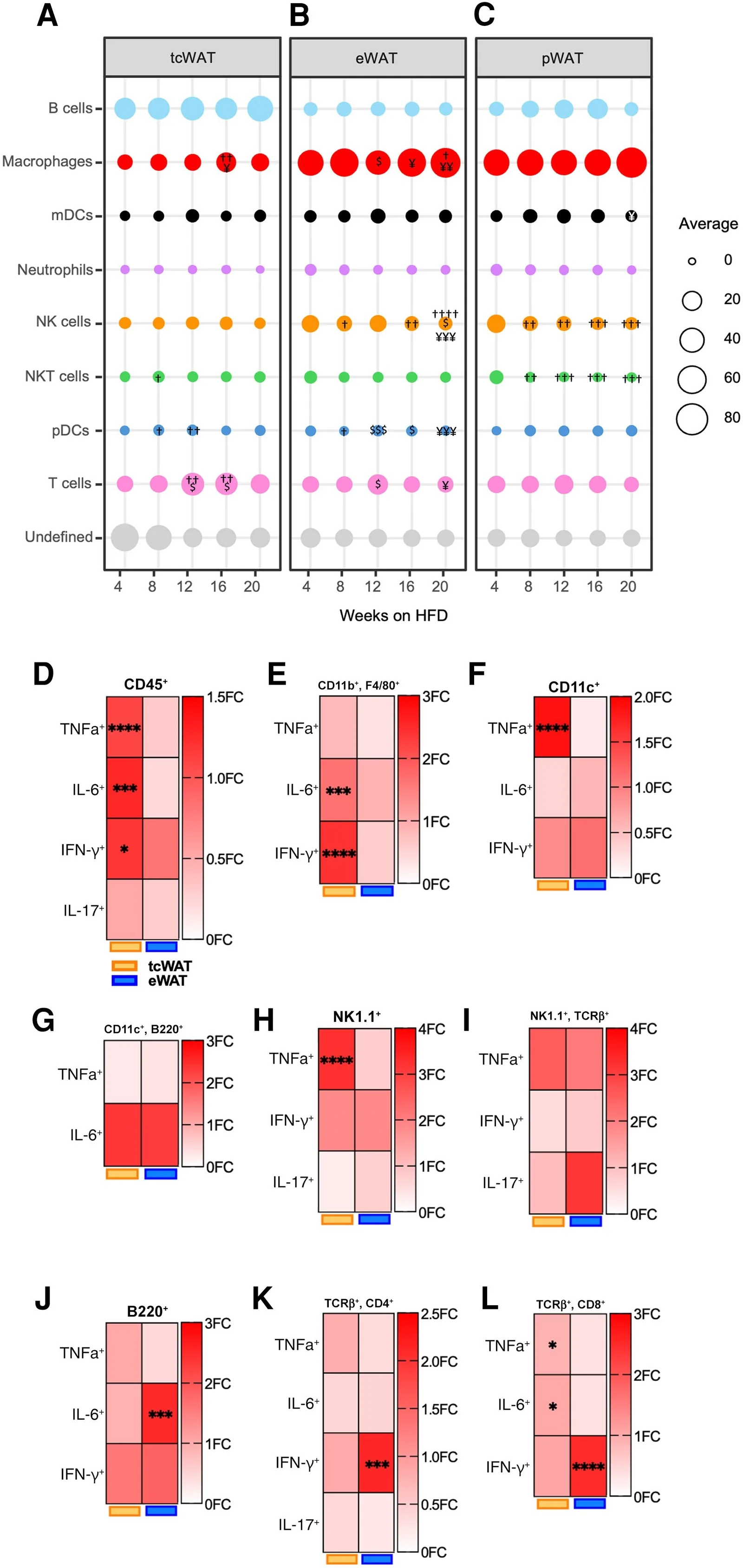
Thoracic cavity WAT harbors a unique and dynamic immune cell composition with enhanced inflammatory nature. Eight-week-old wild-type C57BL/6 mice were fed HFD for 4, 8, 12, 16, 20 weeks. *A–C*, Temporal analysis of immune cell population frequencies in (*A*) tcWAT CD45^+^ cells, (*B*) eWAT CD45^+^ cells, and (*C*) pWAT CD45^+^ cells as determined by flow cytometry. *D–L*, Heatmap of log-fold change of cytokines from 4 weeks to 20 weeks on HFD between tcWAT and eWAT immune cell-derived cytokines: (*D*) CD45^+^, (*E*) CD11b^+^F4/80^+^, (*F*) CD11c^+^, (*G*) CD11c^+^B220^+^, (*H*) NK1.1^+^, (*I*) NK1.1^+^TCRβ^+^, (*J*) B220^+^, (*K*) TCRβ^+^CD4^+^, and (*L*) TCRβ^+^CD8^+^. Data are represented as mean ± SEM. *A–C*, One-way analysis of variance (Dunnett). Cell population data statistics represented as †, significant difference from 4 weeks of HFD; $, significant difference from 8 weeks of HFD; ¥, significant difference from 12 weeks of HFD; 1 symbol *P* < .05, 2 symbols *P* < .01, 3 symbols *P* < .001, 4 symbols *P* < .0001; n = 3–8/group. *D–L*, Student *t* test *P < .05, **P < .01, ***P < .001, ****P < .0001; n = 3–12/group, combined results of 2 independent experiments. Abbreviations: eWAT, epidydimal WAT; FC, fold change; HFD, high-fat diet; IFN-γ, interferon-γ; IL, interleukin; mDC, myeloid dendritic cell; pDC, plasmacytoid dendritic cell; NK cell, natural killer cell; NKT cell, natural killer T cell; pWAT, perirenal WAT; tcWAT, thoracic cavity WAT; TNF-α, tumor necrosis factor-α; WAT, white adipose tissue.

Given heightened immune cell presence and contribution to IAV pathogenesis in obesity, tcWAT immune cell composition was temporally characterized throughout obesity development, with eWAT and pWAT for comparison. Flow cytometric analysis (Supplementary Figure 3*B*) revealed a unique and highly dynamic immune cell composition in tcWAT (Figure 2*A*–*C*). Specifically, tcWAT T-cell receptor β^+^ (TCRβ^+^; T cells) compartment was significantly expanded at 12 and 16 weeks on HFD compared to earlier time points, and was larger than eWAT and pWAT populations (Figure 2*A*–*C*). At 20 weeks of HFD feeding, B220^+^ cells (B cells) were the most populous within tcWAT, becoming almost half of its composition (Figure 2*A*). This directly contrasted with the immune cell composition of eWAT and pWAT, as they were predominantly composed of the CD11b^+^F4/80^+^ (macrophage) population during the entirety of obesity development (Figure 2*B* and *2C*). Histological evaluation of tcWAT was performed next. Notably, the weighted diffuse and overall scores were significantly higher in tcWAT compared to eWAT, confirming its increased lymphocytic presence (Supplementary Figure 1). These data suggest tcWAT harbors a dynamic and lymphocyte-centric immune cell composition.

Obesity-driven skewing toward inflammatory cytokine production systemically and locally [10] is linked to the pathogenesis of obesity-associated diseases [30, 31]. Thus, how obesity modulates tcWAT immune cells’ inflammatory cytokine production compared to other visceral WAT depots was investigated next. Given the absence of tcWAT in a lean state, the early stage of obesity (4 weeks of HFD) was used as a baseline. tcWAT-resident total immune cells (CD45^+^), compared to eWAT counterparts, exhibited robust increases in TNF, IL-6, and interferon-γ (IFN-γ) production (Figure 2*D*). The innate immune cell-associated compartments within tcWAT compared to eWAT produced substantially more IL-6, IFN-γ, or TNF in a cell-specific manner (Figure 2*E*–*H*). The adaptive immune cell-associated compartment also showed differential effects with cell-specific increases or decreases in inflammatory cytokine production (Figure 2*I*–*L*). These data suggest that inflammatory cytokine output differs between tcWAT and eWAT immune cells.

To uncover mechanisms that shape tcWAT immune cell character and function, scRNAseq on tcWAT and eWAT immune cells from obese mice was performed. Cell populations were distinguished by the top 60 marker genes based on ToppCell gene set enrichment (Supplementary Figure 4*A*). Notably, sequencing results displayed tcWAT as lymphocyte dominant, confirming our flow cytometry and histological analyses, a stark contrast to the myeloid-dominant composition of eWAT (Supplementary Figure 4*B*). In fact, the most abundant populations in tcWAT, T Satb1^+^ (a key regulator of T-cell development) and T immature cells, are almost exclusively unique to tcWAT (Supplementary Figure 4*B*). Analysis of differential gene expression in T Satb1^+^ revealed an increase in gene expression associated with chromatin organization and T-cell signaling/activation, and strong decreases in cytoplasmic translation and MHC class I/Ib antigen presentation compared to eWAT (Supplementary Figure 4*C*).

Furthermore, in the tcWAT T Satb1^+^ population we identified *Ets1* as a predicted upregulated transcription factor hub for differentially expressed genes (NetPerspective), upstream of ribosome biogenesis (downregulated), histone binding (upregulated), and T-cell receptor signaling/activation (upregulated). Specifically, these targets include T-cell survival mediator gene, *Stat5b,* and T-cell differentiation genes *Cd4, Tcf7,* and *Rorc* (Supplementary Figure 4*D*). *Ets1* further induced genes indicative of epigenetic remodeling (*Jarid2*, *Ezh2*, *Smarca4*, *Bach1*) (Supplementary Figure 4*D*). These data suggest that tcWAT transcriptional immune landscape is unique, dominated by T Satb1^+^, harboring increased immune regulatory functions.

### Influenza Infection Robustly Modifies Immune Landscape of Thoracic White Adipose Tissue

**Figure 3.**
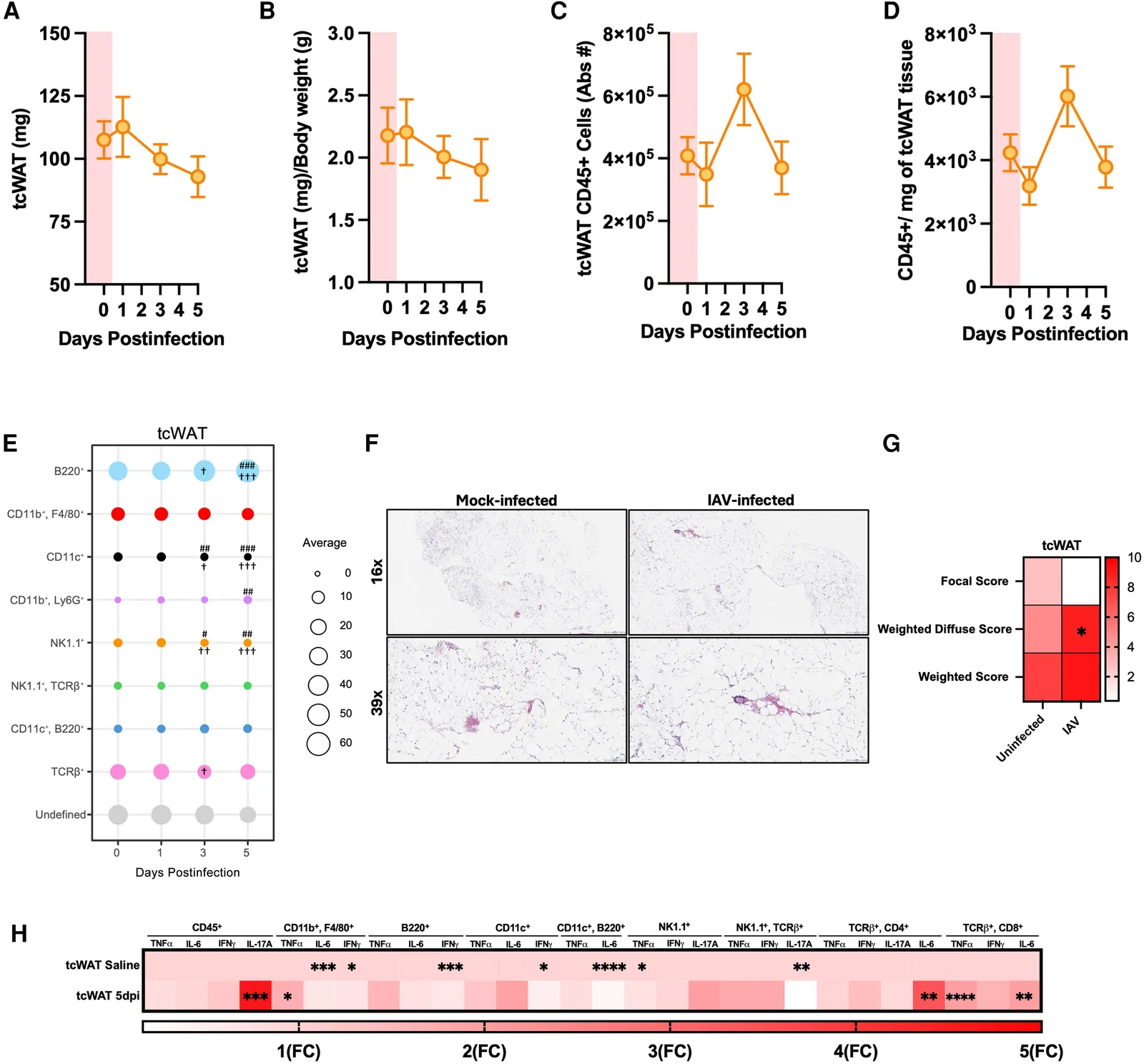
Influenza A virus infection skews the immune profile of tcWAT. Eight-week-old wild-type C57BL/6 mice were fed HFD for 20 weeks and subsequentially mock infected (saline; shaded pink in A-D) or infected with IAV (30 hemagglutinin units). Temporal analysis of (*A*) tcWAT weight; (*B*) tcWAT (mg) per body weight (g); (*C*) absolute numbers of tcWAT CD45^+^ cells via flow cytometry; (*D*) tcWAT CD45^+^ cells/mg of tissue weight; and (*E*) immune cell frequencies of total tcWAT CD45^+^ cells quantified via flow cytometry. *F*, Representative images of uninfected and IAV-infected tcWAT taken at different magnifications, equivalent to 16 × (Mock-infected: 384&micro and 39 × (Mock-infected: 154&micro (hematoxylin and eosin stained). *G*, Focal score ([Ly1 + Ly2 area/total area] × 100); weighted diffuse score (sum of Ly3–Ly5); and weighted score (sum of focal score and weighted diffuse score). *H*, Heatmap of fold changes at 5 dpi relative to mock-infected counterparts of tcWAT CD45^+^, CD11b^+^ F4/80^+^, CD11c^+^, CD11c^+^B220^+^, NK1.1, NK1.1^+^TCRβ^+^, TCRβ^+^CD4^+^, and TCRβ^+^CD8^+^ cell-derived cytokines. Data are represented as mean ± SEM. *A*–*E*, One-way analysis of variance (Dunnett). *G* and *H*, Student *t* test *P < .05, **P < .01, ***P < .001, ****P < .0001. *E*, #, significant difference from mock-infected obese tcWAT; †, significant difference from 1 dpi obese tcWAT; 1 symbol *P* < .05; 2 symbols *P* < .01; 3 symbols *P* < .001. Sample size: (*A*–*D*) n = 10–22, combined result of 4 independent experiments; (*E*) n = 3–25, combined result of 4 independent experiments; (*G*) n = 3–5; (*H*) n = 8–12, combined result of 2 independent experiments. Abbreviations: dpi, days postinfection; FC, fold change; HFD, high-fat diet; IAV, influenza A virus; IFN-γ, interferon-γ; IL, interleukin; tcWAT, thoracic cavity white adipose tissue; TNF-α, tumor necrosis factor-α; Ly, lymphoid.

Given the observed differences in immune cell profile at baseline, we next evaluated the impact of IAV infection on tcWAT immunogenicity. IAV infection induced a trend toward a decrease in tcWAT depot size (Figure 3*A*) and tcWAT/body weight ratio (Figure 3*B*). However, compared to mock-infected counterparts (denoted by day 0), IAV infection did not significantly alter the absolute number (Figure 3*C*) or density of tcWAT immune cells (Figure 3*D*). Notably, while not significant, absolute number and density of immune cells were increased at 3 dpi compared to controls (Figure 3*C* and 3*D*). To investigate which immune cells may contribute to this increase, we conducted flow cytometry analysis throughout IAV infection (1, 3, and 5 dpi). Analysis revealed B220^+^ was the only population significantly increased at 3 dpi compared to 1 dpi counterparts (Figure 3*E*). Conversely, TCRβ^+^, CD11c^+^, and NK1.1^+^ populations were significantly reduced at 3 dpi relative to 1 dpi (Figure 3*E*). At 5 dpi, both B220^+^ and CD11b^+^LyG6^+^ populations were significantly expanded compared to mock-infected controls (Figure 3*E*). Furthermore, 5 dpi CD11c^+^ and NK1.1^+^ populations were decreased relative to both saline and 1 dpi counterparts (Figure 3*E*). The impact of IAV infection on tcWAT histological features was analyzed next. Despite similar overall weighted score between tcWAT from IAV-infected and mock-infected mice, a paucity of lymphocytes in focal aggregating patterns (focal score) and a significant increase in the weighted diffuse score were observed (Figure 3*F* and 3*G*).

The impact of IAV infection on tcWAT immune cell inflammatory cytokine production was examined next. The fold change in cytokine frequencies at 5 dpi, the most advanced stage of infection before mortality begins [24], was calculated using mock-infected values as a control. Among total CD45^+^ cells, IAV infection only significantly amplified IL-17A levels at 5 dpi (Figure 3*H*). Within the innate immune cell-associated populations, CD11b^+^F4/80^+^ had significantly elevated levels of TNF. Additionally, CD11c^+^ cell IL-6 production, NK1.1^+^ cell IL-17A production, and NK1.1^+^TCRβ^+^ cell TNF and IFN-γ production all trended toward an increase after IAV infection (Figure 3*H*). In contrast, IAV infection significantly decreased IFN-γ production among CD11b^+^F4/80^+^ and CD11c^+^ populations (Figure 3*H*). Furthermore, there was a significant reduction in IL-6 among CD11b^+^F4/80^+^ and CD11c^+^B220^+^ populations, along with NK1.1^+^TCRβ^+^ cell IL-17A production (Figure 3*H*). In the adaptive immune cell-associated populations, both TCRβ^+^CD8^+^ and TCRβ^+^CD4^+^ cells had increased levels of IL-6 (Figure 3*H*). Additionally, TCRβ^+^CD8^+^ cells had increased TNF production (Figure 3*H*). However, B220^+^ populations had significantly decreased production of IFN-γ (Figure 3*H*). Next, we examined the changes in the inflammatory potential of tcWAT immune cells during IAV infection. Total tcWAT immune cells from obese mice, mock infected (saline), or IAV infected (5 dpi) were isolated and stimulated ex vivo. Notably, stimulated immune cells had significantly increased production of TNF, IL-6, and IL-17A at 5 dpi compared to mock-infected controls (Supplementary Figure 5*A*–*D*).

### Influenza Infection Expands B Zbtb32^+^ Cells and Antiviral Gene Expression in Thoracic White Adipose Tissue

**Figure 4.**
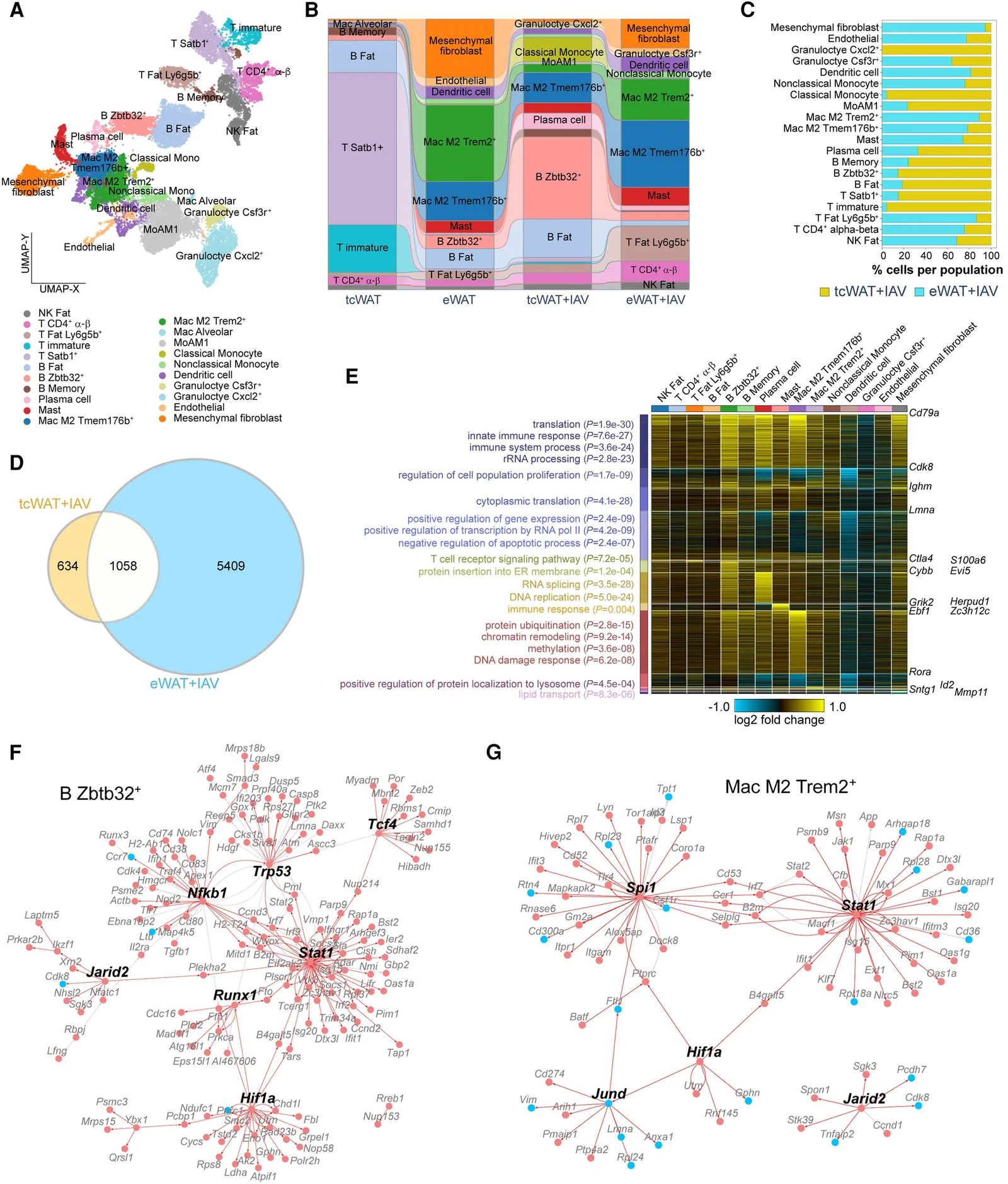
Influenza A virus differentially remodels tcWAT and eWAT. *A*, Integrated scRNAseq UMAP of immune-enriched populations from eWAT, tcWAT, and lung, with and without IAV infection. Scanpy-derived clusters were annotated using prior annotations from lung immune (cellHarmony) and marker genes (ToppFun). *B*, Sankey plot of cell population frequency across mock-infected and IAV-infected cells from WAT. *C*, Comparison of relative frequency between tcWAT + IAV and eWAT + IAV. *D*, Venn diagram of unique differentially expressed genes in any cell state; comparisons for tcWAT and eWAT IAV-infected versus mock-infected, matched by directionality. *E*, Heatmap of differentially expressed genes between any cell population (≥ 25 cells) comparing IAV-infected tcWAT versus IAV-infected eWAT. Rows are genes and columns are log_2_ fold changes. The most significant gene from each cluster is shown on the right and the most enriched GO-terms (GO-Elite) are shown on the left for each cellHarmony gene cluster. *F* and *G*, Predicted gene regulatory network from the software NetPerspective for (*F*) B cells (Zbtb32^+^ cluster) and (*G*) M2 macrophages (Trem2^+^ cluster). Upregulated and down-regulated genes are indicated by red and blue nodes, respectively; red arrows indicate annotated transcriptional regulatory interactions. Abbreviations: eWAT, epidydimal WAT; IAV, influenza A virus; scRNAseq, single-cell RNA sequencing; tcWAT, thoracic cavity WAT; WAT, white adipose tissue.

To compare IAV-infection alterations in the tcWAT immune cell landscape to that of eWAT, we performed scRNAseq on tcWAT and eWAT immune cells from obese IAV-infected mice at 5 dpi (Figure 4*A*). IAV infection induced significant changes in the tcWAT immune cell transcriptome with dominant expansion in B-cell populations (B Zbtb32^+^, plasma cell) and an increase in myeloid populations (classical monocyte, Mac M2 Tmem176^+^, Mac M2 Trem2^+^) (Figure 4*B* and 4*C*). Notably, there was a reduction in T Satb1^+^ and T immature populations (Figure 4*B* and 4*C*). While IAV infection induced drastic changes in the tcWAT immune cell transcriptome, the impact on eWAT was limited (Figure 4*B* and 4*C*). IAV infection-induced tcWAT and eWAT to have mixed lymphocyte and myeloid dominance (Figure 4*B* and 4*C*). Of note, all B-cell populations (plasma cell, B memory, B Zbtb32^+^, B fat) represented a majority of tcWAT composition after IAV infection (Figure 4*B* and 4*C*), supporting our previous flow cytometry results (Figure 3*E*).

Compared to uninfected controls, IAV infection differentially modified 6467 genes in eWAT and 1692 genes in tcWAT, with 1058 genes regulated in the same direction, conserved between the 2 (Figure 4*D*). Comprehensive comparison of tcWAT versus eWAT with IAV infection across all cell populations, with the software cellHarmony [32], identified conserved gene expression impacts across immune populations, primarily denoted by upregulation (Figure 4*E*). Mac M2 Tmem176b^+^ cell upregulated genes were enriched for key mediators of innate immune response genes (eg, *Irf77, Zbp1, Isg15, Rsad2, Ifit1, Oas3*). Upregulated genes in B Zbtb32^+^ cells were enriched for negative regulation of viral genome replication (eg, *Eif2ak2, Isg20, Zc3hav1, St1, Ifh1*) and negative regulation of T-cell proliferation (eg, *Cd274* [PD-L1], *Tgfb1, Il2ra, Cd80, Mad1l1*). To identify presumed drivers of tcWAT transcriptional responses, we again employed transcription factor regulatory prediction using the software NetPerspective [32, 33]. From this analysis, upregulated genes in B Zbtb32^+^ were predicted to be regulated by established immune signaling regulators (*Stat1, Nfkb1*, *Runx1, Tcf3, Tp53,* and *Hif1a*) (Figure 4*F*). *Stat1* directly upregulates various antiviral genes (*Isg15, Is20, Oas1a, Irf7*) and critical lymphocyte differentiator, *Runx1* (Figure 4*F*). Furthermore, *Nfkb1* directly targets *Ccr7*, regulator of immune cell trafficking to secondary lymphoid organs, for downregulation (Figure 4*F*) and upregulates MHC class II antigen presentation genes (*H2-Ab* and *CD74*) (Figure 4*F*). As IAV infection prompted increases in tcWAT myeloid populations, specifically Mac M2 Trem 2^+^, we also investigated its predicted gene interactions during IAV infection. Like the B Zbtb32^+^ population, various immune-related genes were upregulated, including *Spi1*, *Stat1*, *Hif1a*, and *Jund* (Figure 4*G*). *Stat1* similarly targets the upregulation of antiviral genes (*Oas1a, Isg15, Isg20, Irf7*) along with immune cell recruitment gene, *Ccr1* (Figure 4*G*). These data suggest that IAV induces dynamic alterations in the tcWAT immune cell transcriptome and upregulates various immune-related viral defense genes in IAV infection expanded populations.

### Influenza Infection Reduces Neutrophil-Regulating Granulocytes in Thoracic White Adipose Tissue

**Figure 5.**
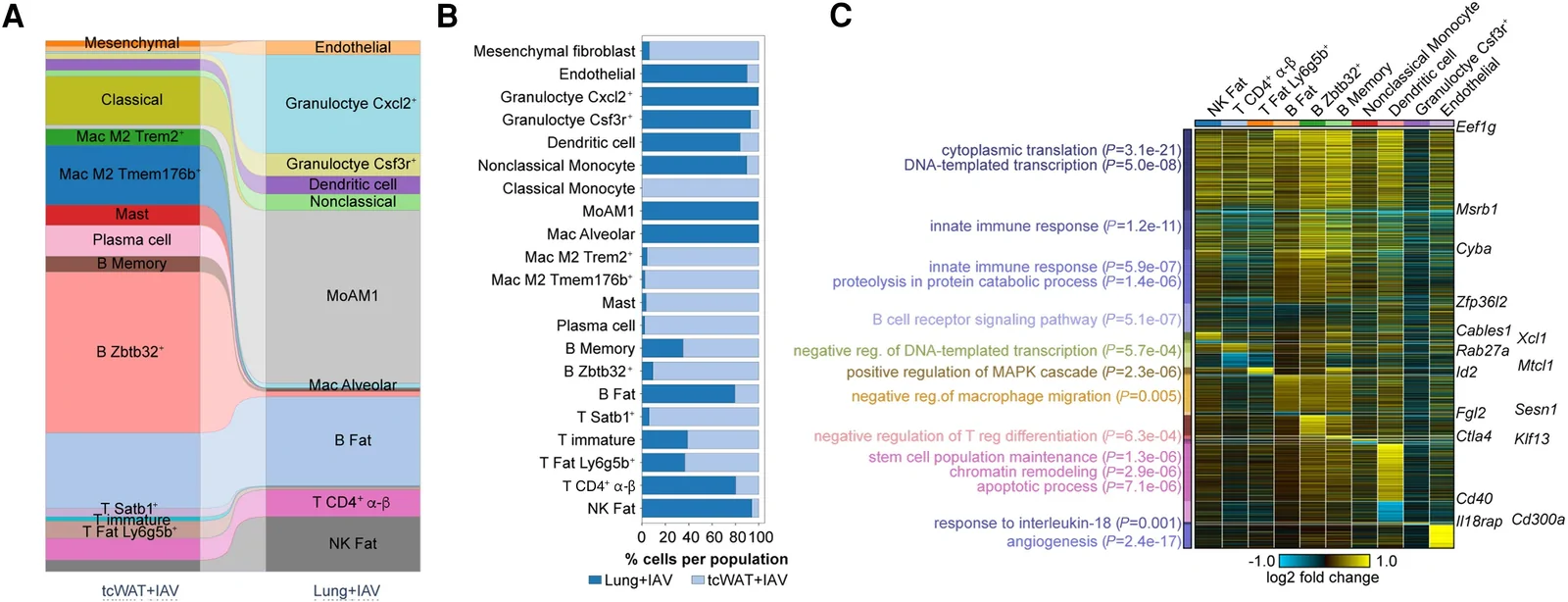
Inverse correlation of granulocyte populations in tcWAT and lung during IAV infection. *A*, Sankey plot of cell population frequency across IAV-infected cells from tcWAT and lung. *B*, Comparison of relative frequency between IAV-infected tcWAT and IAV-infected lung. *C*, Heatmap of differentially expressed genes between any cell population (≥ 25 cells) comparing IAV-infected tcWAT and IAV-infected lung. Abbreviations: IAV, influenza A virus; tcWAT, thoracic cavity white adipose tissue.

We demonstrated transfer of tcWAT immune cells was sufficient to increase host-originated lung immune cell inflammatory cytokine production. To further investigate tcWAT immune cell impact on the lung immune response in IAV infection, we performed scRNAseq on tcWAT and lung immune cells from obese IAV-infected mice at 5 dpi. We found tcWAT and lung immune cell transcriptome compositions were divergent during IAV infection (Figure 5*A*). Specifically, tcWAT immune cell repertoire was more diverse than that of the lung (Figure 5*A*). Notably, one of the lungs most frequent populations was granulocyte Cxcl2^+^ (a chemoattractant for neutrophils), but this population was almost nonexistent in tcWAT (Figure 5*A* and 5*B*). The population granulocyte Csf3r^+^ cells (an essential factor for neutrophil differentiation) was also reduced in tcWAT and increased in the lungs during IAV infection (Figure 5*A* and 5*B*). Given the proximity of tcWAT to the lung, the opposing frequencies of granulocyte Cxcl2^+^ and Csfr3^+^ populations could suggest migration to the lung, which may contribute to the exacerbated IAV infection defense response in obesity.

To investigate the contribution of tcWAT immune cells in exacerbating IAV-infection–induced lung inflammation, we next compared the differential gene expression in tcWAT immune cells to that in lungs during IAV infection. Interestingly, tcWAT B-cell populations (B Zbtb32^+^, B memory, and B fat) had increased gene expression associated with the innate immune response (eg, *Irf7, Trim25, Bst2, Casp4, Cybb, Zc3hav1, Riok3, Btk, Lyn*) and downregulated B-cell receptor signaling (eg, *Cd79b, Syk, Card11, Igkc, Ighm*) (Figure 5*C*). Notably, B Zbtb32^+^ and B memory also had increased gene expression linked to positive regulation of MAPK signaling (eg, *Jak2, Lyn, Ptprj, Bank1, Gadd45b, Adam9*) (Figure 5*C*). Furthermore, the tcWAT granulocyte Csfr3^+^ population had downregulated gene expression associated with innate immune response (eg, *Ifih1, Zbp1, Isg15, Isg20, Oasl1/2, Samhd1, Apobec3*) (Figure 5*C*). Overall, tcWAT immune cells displayed alterations in gene expression that are associated with various immune-related signaling pathways, and this may enhance IAV pathogenesis in obesity by increasing granulocyte populations associated with neutrophil recruitment.

## DISCUSSION

Here, we immunologically characterized tcWAT at baseline and following IAV infection. We demonstrate that tcWAT is a distinct WAT depot enriched in highly inflammatory immune cell populations that are sufficient to accelerate and intensify IAV disease severity. Compared with other visceral WAT depots, tcWAT exhibits a unique immune composition dominated by lymphocytes at baseline, particularly Satb1-expressing T cells with elevated expression of T-cell activation and signaling genes. Following IAV infection, tcWAT undergoes marked immunological remodeling, characterized by expansion of B220^+^ and IL-17A^+^ cells. Transcriptomic analyses revealed a pronounced expansion of the B Zbtb32^+^ population, accompanied by upregulation of pathways associated with immune regulation and antiviral host defense. Additionally, Cxcl2- and Csf3r-expressing granulocyte populations were reduced in tcWAT but increased in the lungs of obese IAV-infected mice, suggesting possible immune cell trafficking from tcWAT to the lung.

We demonstrate tcWAT is largely absent in the lean state (Figure 1*A* and 1*B*). This contrasts with a previous report [23] and likely reflects differences in anatomical definition and tissue collection. In our study, tcWAT was isolated below the lungs to avoid contamination from perithymic WAT. Notably, perithymic WAT is present in lean mice and expands with obesity, but tcWAT's proximity to the thymus and obesity-associated thymic involution increases the likelihood of thymic immune cell contamination [34]. These methodological differences may account for discrepancies regarding tcWAT presence in lean states.

Adoptive transfers demonstrated tcWAT-derived immune cells from IAV-naive donors significantly accelerated mortality and amplified host-derived lung cytokine production in IAV-infected recipients (Figure 1*G*–*J*). Importantly, this effect occurred in the absence of antigen priming, suggesting an antigen-independent mechanism. Cytokine-driven T-cell bystander activation has been implicated in IAV immunopathology [35]. At baseline, tcWAT was enriched with T Satb1^+^ cells exhibiting heightened activation-associated gene expression (Supplementary Figure 4*B* and 4*C*). Following transfer, these cells may be further stimulated by host-derived, IAV-induced inflammatory cytokines, thereby exacerbating disease severity independently of antigen specificity.

A key limitation of the adoptive transfer studies is the limited information on trafficking of tcWAT immune cells posttransfer. Elucidating the migration and tissue localization of these populations is essential to define their mechanism of action. Use of photoconvertible reporter models, such as Kaede mice [36], would allow in vivo tracking of specific tcWAT immune subsets. Of particular interest are Cxcl2^+^ and Csf3r^+^ granulocytes, which were diminished in tcWAT yet enriched in the lungs following IAV infection (Figure 5*A* and 5*B*). These cell types regulate neutrophil recruitment and survival, thus their transfer to the lung may amplify neutrophil-driven inflammation and tissue damage and worsen disease outcomes in obesity.

Our analysis revealed tcWAT immune cell composition was divergent from those in other visceral WAT (Figure 2*A*–*C*, and Supplementary Figure 4*B*). However, tcWAT seemingly defies the conventional dogma of obesity-induced visceral WAT macrophage infiltration, having reduced CD11b^+^F4/80^+^ and other macrophage-associated populations (Figure 2*A* and Supplementary Figure 4*B*). Despite reduced frequency, tcWAT CD11b^+^F4/80^+^ cells produced increased levels of IL-6 and TNF compared to eWAT (Figure 2*E*). Notably, lymphocytes constituted the dominant immune population in tcWAT (Figure 2*A* and Supplementary Figure 4*B*) and exhibited increased proinflammatory cytokine production (Figure 2*L*). Transcriptomic analyses confirmed that Satb1^+^ T cells were the predominant population and displayed enhanced T-cell signaling and activation profiles (Supplementary Figure 4*C*). Collectively, these findings establish tcWAT as a uniquely inflammatory visceral WAT in obesity.

IAV infection induced a robust expansion of B220^+^ cells in tcWAT, driven primarily by the B Zbtb32^+^ subset (Figure 3*E*, and Figure 4*B* and 4*C*). These cells upregulated genes involved in antiviral defense, immune regulation, and antigen presentation, suggesting a potential protective role during primary influenza infection (Figure 4*E* and 4*F*). In B cells, Zbt32 is expressed in a subset of memory cells where it acts as a negative regulator of antibody responses against chronic viral infections (cytomegalovirus) in mice [37]. Considering obesity negatively impacts the IAV antibody response [38] and the tcWAT B Zbt32^+^ population is expanded after IAV infection, an examination of the contribution of B Zbtb32^+^ to disease severity amid reoccurring influenza infections is warranted.

IAV infection increased overall IL-17A production within tcWAT immune cells, despite no clear expansion of canonical IL-17A–producing subsets (Figure 3*H*). As Satb1^+^ T cells are predicted to upregulate *Rorc* expression, it is plausible that these cells differentiate toward a Th17 phenotype during IAV infection (Supplementary Figure 4*D*). Although Satb1^+^ T cells declined during IAV infection, concurrent expansion of CD4^+^ αβ T cells supports this possibility. Future studies should directly evaluate Th17 accumulation and function within tcWAT and their contribution to IAV immunopathology.

Mouse eWAT and pWAT correspond to human visceral WAT depots implicated in immune responses and disease severity [39]. Notably, tcWAT in mice corresponds to mediastinal fat in humans [40]. Given the impact of WAT in influenza disease severity in mice, future analyses of the contribution of mediastinal fat in IAV control in individuals with severe obesity is warranted.

## Supplementary Material

jiag201_Supplementary_Data

## Supplementary Data

Supplementary materials are available at *The Journal of Infectious Diseases* online (http://jid.oxfordjournals.org/). Supplementary materials consist of data provided by the author that are published to benefit the reader. The posted materials are not copyedited. The contents of all supplementary data are the sole responsibility of the authors. Questions or messages regarding errors should be addressed to the author.

## References

[1] World Health Organization . Influenza (seasonal). 2025. https://www.who.int/news-room/fact-sheets/detail/influenza-(seasonal). Accessed 7 October 2025.

[2] Zhang K, Huang Q, Li X, et al The cGAS-STING pathway in viral infections: a promising link between inflammation, oxidative stress and autophagy. Front Immunol 2024; 15:1352479. 10.3389/fimmu.2024.1352479 PMC10902852 38426093

[3] Qi Y, Wu Z, Chen D, Zhu L, Yang Y. A role of STING signaling in obesity-induced lung inflammation. Int J Obes (Lond) 2023; 47:325–34. 10.1038/s41366-023-01272-x PMC9924210 36782056

[4] Califano D, Furuya Y, Roberts S, Avram D, McKenzie ANJ, Metzger DW. IFN-gamma increases susceptibility to influenza A infection through suppression of group II innate lymphoid cells. Mucosal Immunol 2018; 11:209–19. 10.1038/mi.2017.41 PMC5693789 28513592

[5] World Health Organization . Obesity and overweight. 2025. https://www.who.int/news-room/fact-sheets/detail/obesity-and-overweight#:∼:text=In%202022%2C%201%20in%208,million%20were%20living%20with%20obesity. Accessed 26 February 2025.

[6] Writing Committee of the WHO Consultation on Clinical Aspects of Pandemic (H1N1) 2009 Influenza . Clinical aspects of pandemic 2009 influenza A (H1N1) virus infection. N Engl J Med 2010; 362:1708–19. 10.1056/NEJMra1000449 20445182

[7] Pugliese G, Liccardi A, Graziadio C, Barrea L, Muscogiuri G, Colao A. Obesity and infectious diseases: pathophysiology and epidemiology of a double pandemic condition. Int J Obes (Lond) 2022; 46:449–65. 10.1038/s41366-021-01035-6 35058571

[8] Zhao X, Gang X, He G, et al Obesity increases the severity and mortality of influenza and COVID-19: a systematic review and meta-analysis. Front Endocrinol (Lausanne) 2020; 11:595109. 10.3389/fendo.2020.595109 PMC7779975 33408692

[9] Honce R, Schultz-Cherry S. Impact of obesity on influenza A virus pathogenesis, immune response, and evolution. Front Immunol 2019; 10:1071. 10.3389/fimmu.2019.01071 PMC6523028 31134099

[10] Uti DE, Atangwho IJ, Omang WA, et al Cytokines as key players in obesity low grade inflammation and related complications. Obesity Med 2025; 54:100585.

[11] Paich HA, Sheridan PA, Handy J, et al Overweight and obese adult humans have a defective cellular immune response to pandemic H1N1 influenza A virus. Obesity (Silver Spring) 2013; 21:2377–86. 10.1002/oby.20383 PMC3695020 23512822

[12] Frayn KN, Karpe F, Fielding BA, Macdonald IA, Coppack SW. Integrative physiology of human adipose tissue. Int J Obes Relat Metab Disord 2003; 27:875–88. 10.1038/sj.ijo.0802326 12861227

[13] Vazquez-Vela ME, Torres N, Tovar AR. White adipose tissue as endocrine organ and its role in obesity. Arch Med Res 2008; 39:715–28. 10.1016/j.arcmed.2008.09.005 18996284

[14] Chan CC, Damen M, Alarcon PC, Sanchez-Gurmaches J, Divanovic S. Inflammation and immunity: from an adipocyte's perspective. J Interferon Cytokine Res 2019; 39:459–71. 10.1089/jir.2019.0014 PMC6660836 30920343

[15] Sun K, Kusminski CM, Scherer PE. Adipose tissue remodeling and obesity. J Clin Invest 2011; 121:2094–101. 10.1172/JCI45887 PMC3104761 21633177

[16] Borgeson E, Boucher J, Hagberg CE. Of mice and men: pinpointing species differences in adipose tissue biology. Front Cell Dev Biol 2022; 10:1003118. 10.3389/fcell.2022.1003118 PMC9521710 36187476

[17] Alarcon PC, Ulanowicz CJ, Damen MSMA, et al Obesity uncovers presence of inflammatory lung macrophage subsets with adipose tissue transcriptomic signature in influenza virus infection. J Infect Dis 2024; 231:e317–27. 10.1093/infdis/jiae535 PMC11841630 39494998

[18] Ayari A, Rosa-Calatrava M, Lancel S, et al Influenza infection rewires energy metabolism and induces browning features in adipose cells and tissues. Commun Biol 2020; 3:237. 10.1038/s42003-020-0965-6 PMC7224208 32409640

[19] Khan S, Chan YT, Revelo XS, Winer DA. The immune landscape of visceral adipose tissue during obesity and aging. Front Endocrinol (Lausanne) 2020; 11:267. 10.3389/fendo.2020.00267 PMC7243349 32499756

[20] Sárvári AK, Van Hauwaert EL, Markussen LK, et al Plasticity of epididymal adipose tissue in response to diet-induced obesity at single-nucleus resolution. Cell Metab 2021; 33:437–53.e435. 10.1016/j.cmet.2020.12.004 33378646

[21] Nishimura H, Itamura S, Iwasaki T, Kurata T, Tashiro M. Characterization of human influenza A (H5N1) virus infection in mice: neuro-, pneumo- and adipotropic infection. J Gen Virol 2000; 81:2503–10. 10.1099/0022-1317-81-10-2503 10993940

[22] Barthelemy J, Bogard G, Wolowczuk I. Beyond energy balance regulation: the underestimated role of adipose tissues in host defense against pathogens. Front Immunol 2023; 14:1083191. 10.3389/fimmu.2023.1083191 PMC10019896 36936928

[23] Hornung F, Schulz L, Köse-Vogel N, et al Thoracic adipose tissue contributes to severe virus infection of the lung. Int J Obes (Lond) 2023; 47:1088–99. 10.1038/s41366-023-01362-w PMC10599992 37587162

[24] Alarcon PC, Damen MSMA, Ulanowicz CJ, et al Obesity amplifies influenza virus-driven disease severity in male and female mice. Mucosal Immunol 2023; 16:843–58. 10.1016/j.mucimm.2023.09.004 PMC10842771 37730122

[25] Moreno-Fernandez ME, Giles DA, Oates JR, et al PKM2-dependent metabolic skewing of hepatic Th17 cells regulates pathogenesis of non-alcoholic fatty liver disease. Cell Metab 2021; 33:1187–204.e1189. 10.1016/j.cmet.2021.04.018 PMC8237408 34004162

[26] Chan CC, Damen MSMA, Moreno-Fernandez ME, et al Type I interferon sensing unlocks dormant adipocyte inflammatory potential. Nat Commun 2020; 11:2745. 10.1038/s41467-020-16571-4 PMC7265526 32488081

[27] Hui L, Li Y, Huang MK, Jiang YM, Liu T. CXCL13: a common target for immune-mediated inflammatory diseases. Clin Exp Med 2024; 24:244. 10.1007/s10238-024-01508-8 PMC11499446 39443356

[28] Liu M, Guo S, Hibbert JM, et al CXCL10/IP-10 in infectious diseases pathogenesis and potential therapeutic implications. Cytokine Growth Factor Rev 2011; 22:121–30. 10.1016/j.cytogfr.2011.06.001 PMC3203691 21802343

[29] Dong Y, Dong Y, Zhu C, et al Targeting CCL2-CCR2 signaling pathway alleviates macrophage dysfunction in COPD via PI3K-AKT axis. Cell Commun Signal 2024; 22:364. 10.1186/s12964-024-01746-z PMC11253350 39014433

[30] Huang LY, Chiu CJ, Hsing CH, Hsu YH. Interferon family cytokines in obesity and insulin sensitivity. Cells 2022; 11:4041. 10.3390/cells11244041 PMC9776768 36552805

[31] Mills KHG . IL-17 and IL-17-producing cells in protection versus pathology. Nat Rev Immunol 2023; 23:38–54. 10.1038/s41577-022-00746-9 PMC9255545 35790881

[32] DePasquale EAK, Schnell D, Dexheimer P, et al cellHarmony: cell-level matching and holistic comparison of single-cell transcriptomes. Nucleic Acids Res 2019; 47:. 10.1093/nar/gkz789 PMC6868361 31529053

[33] McLendon PM, Davis G, Gulick J, et al An unbiased high-throughput screen to identify novel effectors that impact on cardiomyocyte aggregate levels. Circ Res 2017; 121:604–16. 10.1161/CIRCRESAHA.117.310945 PMC5581213 28655832

[34] Yang H, Youm Y-H, Vandanmagsar B, et al Obesity accelerates thymic aging. Blood 2009; 114:3803–12. 10.1182/blood-2009-03-213595 PMC2773495 19721009

[35] Shim CH, Cho S, Shin YM, Choi JM. Emerging role of bystander T cell activation in autoimmune diseases. BMB Rep 2022; 55:57–64. 10.5483/BMBRep.2022.55.2.183 PMC8891623 35000675

[36] Tomura M, Yoshida N, Tanaka J, et al Monitoring cellular movement in vivo with photoconvertible fluorescence protein “kaede” transgenic mice. Proc Natl Acad Sci U S A 2008; 105:10871–6. 10.1073/pnas.0802278105 PMC2504797 18663225

[37] Jash A, Zhou YW, Gerardo DK, et al ZBTB32 restrains antibody responses to murine cytomegalovirus infections, but not other repetitive challenges. Sci Rep 2019; 9:15257. 10.1038/s41598-019-51860-z PMC6813321 31649328

[38] Abd Alhadi M, Friedman LM, Karlsson EA, et al Obesity is associated with an impaired baseline repertoire of anti-influenza virus antibodies. Microbiol Spectr 2023; 11:e0001023. 10.1128/spectrum.00010-23 PMC10269616 37098954

[39] Ziegler AK, Scheele C. Human adipose depots’ diverse functions and dysregulations during cardiometabolic disease. NPJ Metab Health Dis 2024; 2:34. 10.1038/s44324-024-00036-z PMC11606922 39619657

[40] Cheung L, Gertow J, Werngren O, et al Human mediastinal adipose tissue displays certain characteristics of brown fat. Nutr Diabetes 2013; 3:e66. 10.1038/nutd.2013.6 PMC3671748 23670224

