# Supplementary material for: Distinct Inflammatory Programming of Thoracic Cavity White Adipose Immune Cells Regulates Influenza Pathogenesis
Source: J Infect Dis. 2026 Apr 7;234(3):e541–51. doi: 10.1093/infdis/jiag201 (PMC13419038; doi:10.1093/infdis/jiag201)
Supplement: Supplementary file 1 [file jiag201_supplementary_data.zip › Ulanowicz_Supplemental Figures_tcWAT+IAV.docx]

**Supplemental Figure 1: Prominent and varying lymphocytic patterns in thoracic cavity white adipose.**

Representative image of tcWAT H&E stained non-inflamed and hypertrophic adipocytes. (**B**) Lymphocytic pattern 1 (Ly1). (**C**) Lymphocytic pattern 2 (Ly2). (**D**) Lymphocytic pattern 3 (Ly3). (**E**) Lymphocytic pattern 4 (Ly4). (**F**) Representative image of tcWAT and eWAT taken different magnifications, equivalent to 16x and 39x objectives (scale bars in micrometers; H&E stained). (**G**) Focal score: ((Ly1+Ly2 area/total area)* 100)); Weighted Diffuse Score (sum of Ly3-Ly5); Weighted Score (sum of Ly1-Ly5). n = 3-4/group. Data are represented as mean ± SEM. Student *t* test. *P < .05.

**Supplementary Figure 2: High fat diet (HFD)-feeding promotes immune cell accrual across WAT depots.**

8-week-old WT C57BL/6 male mice were fed CD or HFD for 4, 8, 12, 16, 20 weeks. Total immune cells (CD45^+^) were quantified by flow cytometry. (**A**) Left panel: CD45^+^ cells in tcWAT across the duration of HFD-feeding; Right panel: tcWAT CD45^+^ cells per mg of tissue. (**B**) Left panel: CD45^+^ cells in eWAT across the duration of HFD-feeding; Right panel: eWAT CD45^+^ cells per mg of tissue. (**C**) Left panel: CD45^+^ cells in pWAT across the duration of HFD-feeding; Right panel: pWAT CD45^+^ cells per mg of tissue. Data are represented as mean ± SEM. 1-way analysis of variance. Statistics represented as #: significant difference from 0 weeks of HFD feeding. †: significant difference from 4 weeks of HFD feeding; $: significant difference from 8 weeks of HFD feeding; ¥ significant difference from 12 weeks of HFD feeding; ^ significant difference from 16 weeks of HFD feeding. Number of symbols represents power of p-value. 1 symbol P < .05. 3 symbols P<0.001. n = 2-12/group; combined result of 2 independent experiments.

**Supplemental Figure 3: Flow gating strategy for identification of immune cell populations.**

Representative schematic of gating strategy used to identify various immune cell populations. Lymphocytes gated on SSC-A vs FSC-A. Live cells gated within that population via SSC-A vs Zombie UV. CD45^+^ cells gated within live cells via SSC-A vs CD45^+^. All following cell populations gated within CD45^+^ population. Presumed neutrophils gated via CD11b^+^Ly6G^+^. Presumed macrophages gated via CD11b^+^F4/80^+^. Presumed mDCs gated via CD11C^+^. Presumed pDCs gated via CD11c^+^B220^+^. Presumed B cells gated via B220^+^. Presumed NK cells gated via NK1.1^+^. Presumed NKT cells gated via NK1.1^+^TCRβ^+^. Presumed T cells gated via TCRβ^+^. Presumed CD4^+^ T cells gated via TCRβ^+^CD4^+^. Presumed CD8^+^ T cells gated via TCRβ^+^CD8^+^.

**Supplemental Figure 4. Distinct immune subsets and lymphoid transcriptional states in thoracic cavity white adipose.**

Heatmap of the top marker genes for scanpy defined clusters from integrated white adipose and lung scRNA-Seq captures, mock-infected or IAV infection. (**B**) Comparison of relative frequency between mock-infected tcWAT and eWAT depots. (**C**) Heatmap of differentially expressed genes between any cell population (≥ 25 cells) comparing mock-infected tcWAT versus eWAT. (**D**) Predicted gene regulatory network from the software NetPerspective for T cells (Satb1+ cluster). Upregulated and down-regulated genes are indicated by red and blue nodes, respectively. Red arrows indicate annotated transcriptional regulatory interactions.

**Supplemental Figure 5: Increased inflammatory potential by thoracic cavity white adipose immune cells during influenza infection.**

8-week-old WT C57BL/6 male mice were fed HFD for 20 weeks and subsequentially mock-infected or infected with IAV (30HA). tcWAT immune cell (CD45^+^) cytokine production was quantified via flow cytometry. Cells were stimulated *ex vivo* with PMA and Ionomycin for 4 hours. (A) IL-6 production in IAV infected compared to mock-infected counterparts. (B) TNF production in IAV infected compared to mock-infected counterparts. (C) IL-17A production in IAV infected compared to mock-infected counterparts. Data are represented as mean ± SEM. Student *t* test. **P < .01. ***P < .001. n = 7-13/group; combined result of 2 independent experiments.
