# Supplementary material for: Distinct Inflammatory Programming of Thoracic Cavity White Adipose Immune Cells Regulates Influenza Pathogenesis
Source: J Infect Dis. 2026 Apr 7;234(3):e541–51. doi: 10.1093/infdis/jiag201 (PMC13419038; doi:10.1093/infdis/jiag201)
Supplement: Supplementary file 1 [file jiag201_supplementary_data.zip › Ulanowicz_Supplemental Table 1_tcWAT+IAV.docx]

**Supplemental Table 1: qRT-PCR Genes and Sequences**

| Gene Name | Sequence |
| --- | --- |
| *Cxcl13* Sense | 5ʹ- GGC CAC GGT ATT CTG GAA GC -3ʹ |
| *Cxcl13* Protein Antisense | 5ʹ- GGG CGT AAC TTG AAT CCG ATC TA -3ʹ |
| *Cxcl10* Sense | 5’- CCT ATG GCC CTC ATT CTC AC -3’ |
| *Cxcl10* Antisense | 5’- CGT CAT TTT CTG CCT CAT CC -3’ |
| *Cxcl9* Sense | 5’- TAG GCA GGT TTG ATC TCC GT -3’ |
| *Cxcl9* Antisense | 5’- CGA TCC ACT ACA AAT CCC TCA -3’ |
| *Ccl2* Sense | 5’- TGC AGT TAA CGC CCC AC -3’ |
| *Ccl2* Antisense | 5’- TGT CTG GAC CCA TTC CTT CTT G -3’ |
| *Ccl3* Sense | 5’- ACC ATG ACA CTC TGC AAC CAA G -3’ |
| *Ccl3* Antisense | 5’- TTG GAG TCA GCG CAG ATC TG -3’ |
| *Ccl4* Sense | 5’- TCT CTC CTC TTG CTC GTG GC -3’ |
| *Ccl4* Antisense | 5’- GAA TAC CAC AGC TGG CTT GGA -3’ |
| *β-actin* Sense | 5’- GGC CCA GAG CAA GAG AGG TA G -3’ |
| *β-actin* Antisense | 5’- GGT TGG CCT TAG GTT TCA GG -3’ |
