# Supplementary material for: Distinct Inflammatory Programming of Thoracic Cavity White Adipose Immune Cells Regulates Influenza Pathogenesis
Source: J Infect Dis. 2026 Apr 7;234(3):e541–51. doi: 10.1093/infdis/jiag201 (PMC13419038; doi:10.1093/infdis/jiag201)
Supplement: Supplementary file 1 [file jiag201_supplementary_data.zip › Ulanowicz_Supplementary Methods_tcWAT+IAV_revised.docx]

**Supplementary File: Methods**

All animal care and procedures provided were in accordance with the Guide for the Care and Use of Laboratory Animals and follow NIH regulations, as outlined in DHHS publication Guide for the Care and Use of Laboratory Animals, the Animal Welfare Act (H12336, 12/7/85) and other relevant federal and local laws.

***Obesogenic Diet Model***

C57BL/6J WT mouse breeding pairs were purchased from Jackson Laboratories (Bar Harbor, ME, USA). All mice were housed and bred at Cincinnati Children’s Hospital Medical Center (CCHMC) specific pathogen-free (spf) facility that maintains automatic 10 h dark/14 h light cycle and 22°C temperature, with free access to autoclaved food and water. 6-to 8-week-old male mice were fed an irradiated high fat diet (HFD; fat 60% kcal, Research Diets #D12492) or chow diet (CD; LAB Diet #5010; calories provided by carbohydrates [58%], fat [13%], and protein [29%]). Body weight and food consumption were recorded weekly. All animal care and procedures provided were in accordance with the Guide for the Care and Use of Laboratory Animals and follow NIH regulations, as outlined in DHHS publication Guide for the Care and Use of Laboratory Animals, the Animal Welfare Act (H12336, 12/7/85) and other relevant federal and local laws.

***Influenza Infections***

Influenza infections were carried out as previously described^25,30^. Briefly, mice were sedated via isoflurane inhalation (2.5% isoflurane; Akorn, NDC, 59399-106-01) and treated intranasally with saline (50μL, veterinary grade; Hospira, NDC, 0409-4888-02) or H1N1 PR8 influenza (30HA Units/7.16 log_10_TCID_50_ in 50μL of veterinary grade saline; Charles River: Influenza A/PR/8/34, Batch: 4XP160913). Mice were monitored (weight measured daily) before tissue collection at 1-, 3- or 5-days post infection. In accordance with IACUC regulations, weight loss exceeding 30% of their body weight due to influenza infection were removed from the study.

***Quantitative Reverse Transcriptase-Polymerase Chain Reaction***

White adipose tissue (WAT) samples were homogenized in TRIzol (Invitrogen) using a TissueLyser (Qiagen). RNA was extracted according to manufacturer instructions and reverse-transcribed to cDNA (Verso cDNA Synthesis Kit; Thermo Scientific, Waltham, MA, USA). Quantitative polymerase chain reaction was completed using Qs7 Flex (Applied Biosystems) according to manufacturer’s instructions. Primer sequences used:

***Immune Cell Isolation***

WAT immune cells were isolated using enzymatic digestion as previously described^25,31,32^. Briefly, WAT was dissected, cut into small pieces, and incubated at 37°C while shaking (220 RPM) in digestion buffer containing Liberase TM (21 μg/mL, Roche), DNAse I (8.8 μg/mL, Roche) in Dulbecco’s modified Eagle’s medium (7 mL/sample, DMEM; Corning) with 1% HEPES (Gibco) and 2% bovine serum albumin (Sigma), for 45 minutes. After digestion cells were filtered through a 100μM strainer (VWR) and centrifuged at 800 x g for 8 min, followed by red blood cells lysis.

Lung immune cells were isolated using enzymatic digestion as previously described^25,30^. Briefly, lungs were dissected, cut into small pieces, and incubated at 37°C (5% CO_2_) in the digestion buffer described above for 45 minutes. After digestion, lungs were manually dissociated through a 70μM cell strainer (VWR) and centrifuged at 2000 RPM for 4 min, followed by red blood cell lysis.

***Flow Cytometry***

Single cell suspensions of immune cells from WAT and lungs were used to determine immune cell populations and their ability to produce cytokines as previously described^25,30,31^. For characterization of immune cell populations and baseline cytokine production, cells were stained with Live/dead (Zombie UV Dye, BioLegend) and with directly conjugated monoclonal antibodies to CD45-AF700 or -PEDazzle (BioLegend, 30-F11; BioLegend, 104), TCRβ-APCef780 (BioLegend, 17A2), CD4-FITC (BioLegend, GK1.5), CD8-BV510 (BioLegend, 53-6.7), CD11b-eFluor450 (e-Biosciences, M1/70), F4/80-APC or -AF700 (e-Biosciences, BM8; e-Biosciences, BM8) Ly6G-BV605 (BioLegend, 1A8), B220-BV785 or -BV605 (BioLegend, RA3-6B2; BioLegend, RA3-6B2) CD11c-BV711 (BioLegend, N418), NK1.1-PEDazzle or -FITC (BioLegend, PK136; BioLegend, PK136), then fixed, permeabilized, and stained with directly conjugated antibodies to IL-6-PE (e-Biosciences, MP5-20F3), TNFα-BV650 (BioLegend, MP6-XT22) IFNγ-PeCy7 (e-Biosciences, XMG1.2), IL-17A-Percpcy5.5 (e-Biosciences, 17B7). For quantification of immune cells ability to produce cytokines, single cell suspensions were treated for 4 hours with Phorbol 12-myristate 13-acetate (PMA; 50 ng/mL; Sigma-Aldrich), Ionomycin (1 μg/mL; EMD Millipore), and Brefeldin A (10 mg/mL; GoldBio). All data was collected using an LSR Fortessa flow cytometer (BD Biosciences) and analyzed using FlowJo X software (v10).

***Histopathological Analysis***

Sections of eWAT and tcWAT were fixed in 10% buffered formalin and stained with H&E, per routine clinical practices. Histological scoring of immune cell infiltration was performed on H&E, by an experienced senior certified pathologist, scoring developed on the basis of patterns seen in standard practice. Factors evaluated in scoping include the type of the inflammatory cell population, (density of aggregates and areas they occupy, extent and distribution patterns,) as well as background fat characteristics (normal vs estimated hypertrophy vs damage/fat necrosis.)  Patterns were described as followed: Ly1 (**Figure S1B**) and Ly2 (**Figure S1C**) pertain to tight lymphocyte aggregates and how they infiltrate the tissue, focal score is the average of these 2 patterns. Ly3 (**Figure S1D**) refers to lymphocytes present in a distinct perivascular sheath and Ly4 (**Figure S1E**) references those found pericellular (surrounding stellate microaggregates). Lastly, Ly5 (**Figure S1F**) refers to lymphocytes minimally infiltrating the interstitial space. The absence of a lymphocytic pattern in thoracic cavity WAT is represented in **Figure S1A**. The weighted diffuse score is quantified based of the sums of Ly3, Ly4 and Ly5 pattern scores. The overall weighted score pertains to the sum of the focal score and weighted diffuse score. Representative sections of tcWAT and eWAT display their cellular concentration differences (**Figure S1G**).

***Single-Cell RNA Sequencing and Analysis***

We performed scRNA-seq on 5-10,000 targeted CD45+ immune cells isolated by fluorescence-activated cell sorting from tcWAT, eWAT and lung using the 10x Genomics Chromium 3 ' version 3.1 protocol on a Chromium v2 Controller. Resultant demultiplexed FASTQ files were aligned to the murine transcriptome (10x Genomics mm10-2.1.0 reference) with CellRanger version 7.0.1 with intron-mode. Ambient RNA correction was performed using the SoupX workflow (15% correction), on the CellRanger produced filtered and raw feature matrix files. Quality control (mit_percent 25 --min_genes 200 --min_counts 500) and unsupervised clustering was performed using scanpy (leiden resolution 0.5) through the automated cellHarmony_lite function in AltAnalyze version 3 (https://github.com/SalomonisLab/altanalyze3). MarkerGenes were produced using the AltAnalyze3 marker_heatmap_h5ad function using the MarkerFinder algorithm. Sankey plots were generated using the AltAnalyze3 sankey_h5ad function. Marker gene set enrichment was performed with ToppFun, using the ToppCell marker database. Differential expression between each scRNA-Seq condition was performed using the AltAnalyze3 cellHarmony_differential function (fold>1.2 and Wilcoxon rank-sum test p<0.05, with Benjamini-Hochberg FDR correction). This function calls the NetPerspective function of AltAnalyze to infer gene regulatory networks from annotated transcription factor-target interactions (PAZAR, TRRUST, Amadeus, WikiPathways, KEGG). Gene set enrichment of cellHarmony differentially expressed are computed with the goelite module of AltAnalyze3, using default parameters. The scRNAseq data sets have been deposited in the Gene Expression Omnibus Database (accession GSE266326).

***Immune Cell Adoptive Transfers***

The adoptive transfer of WAT immune cells was done as previously described^25^. Briefly, immune cells were isolated from epididymal and thoracic cavity WAT depots and enriched via flow cytometric cell sorting. 5x10^5^ sorted WAT immune cells were transferred via tail vein injection into recipient obese mice that had been infected with H1N1 PR8 influenza virus 2 days prior.

***Statistical Analysis***

For statistical analysis, a normal distribution was assumed unless noted otherwise, and parametric tests were used. For comparisons between 2 groups an unpaired Student’s t-test (non-categorical) was used. For comparisons between three or more groups with one independent variable a one-way ANOVA (non-categorical) was employed, and with two independent variables, a two-way ANOVA (non-categorical) was employed. One-way ANOVA using Dunnett’s post hoc test and Two-way ANOVA using Tukey’s post hoc test to assess differences between specific groups. Statistical analysis was completed using Prism 10 (GraphPad Software). All values are represented as mean ± standard error mean (SEM). Power analysis to determine the sample size was not performed. The sample size in each study was based on previous studies. Only histological analyses/quantification were conducted in blinded fashion.
