# Supplementary material for: Distinct Inflammatory Programming of Thoracic Cavity White Adipose Immune Cells Regulates Influenza Pathogenesis
Source: J Infect Dis. Author manuscript; Available in PMC 2026 Jul 29. (PMC13419038; doi:10.1093/infdis/jiag201)
Supplement: Supplemental Table 2 [file NIHMS2182055-supplement-Supplemental_Table_2.docx]

**Supplemental Table 2: Flow Cytometry Markers and Antibodies**

General Panel

| **Marker** | **Antibody** |
| --- | --- |
| Live/dead | Zombie UV Dye (BioLegend) |
| CD45 | AF700 (BioLegend, clone 30-F11) or PEDazzle (BioLegend, clone 30-F11) |
| TCRβ | APCef780 (BioLegend, clone 17A2) |
| CD4 | FITC (BioLegend, clone GK1.5) |
| CD8 | BV510 (BioLegend, clone 53-6.7) |
| CD11b | eFluor450 (eBioscience, clone M1/70) |
| CD11c | BV711 (BioLegend, clone N418) |
| NK1.1 | PEDazzle (BioLegend, clone PK136) or FITC (BioLegend, clone PK136) |
| F4/80 | APC eBiosciences, clone BM8) or AF700 (eBiosciences, clone BM8) |
| Ly6G | BV605 (BioLegend, clone 1A8) |
| B220 | BV785 BioLegend, clone RA3-6B2) or BV605 (BioLegend, clone RA3-6B2) |
| IFNγ | PeCy7 (eBiosciences, clone XMG1.2) |
| IL6 | PE (eBioscience, clone MP5-20F3) |
| IL-17A | Percpcy5.5 (eBiosciences, clone 17B7). |
| TNFα | BV650 (BioLegend, clone MP6-XT22) |

Adoptive Transfer Study Panel

| Live/dead | Zombie UV Dye (BioLegend) |
| --- | --- |
| CD45.1 | PE (eBioscience, clone A20) |
| CD45.2 | FITC (eBioscience, clone 104) |
| F4/80 | APC (eBioscience, clone BM8) |
| CD11b | eFluor450 (eBioscience, clone M1/70) |
| Ly6G | BV605 (BioLegend, clone 1A8) |
| TCRβ | APCef780 (BioLegend, clone 17A2) |
| CD8 | BV510 (BioLegend, clone 53-6.7) |
| B220 | BV785 BioLegend, clone RA3-6B2) |
| NK1.1 | AF700 (BioLegend, clone PK136) |
| CD11c | BV711 (BioLegend, clone N418) |
| TNFα | BV650 (BioLegend, clone MP6-XT22) |
| IFNγ | PeCy7 (eBiosciences, clone XMG1.2) |
| IL-6 | PerCP-eFluor710 (eBiosciences, clone MP5-20F3) |
